# Knockin’ on Egg’s Door: Maternal Control of Egg Activation That Influences Cortical Granule Exocytosis in Animal Species

**DOI:** 10.3389/fcell.2021.704867

**Published:** 2021-09-03

**Authors:** Japhet Rojas, Fernando Hinostroza, Sebastián Vergara, Ingrid Pinto-Borguero, Felipe Aguilera, Ricardo Fuentes, Ingrid Carvacho

**Affiliations:** ^1^Laboratorio Fisiología de la Reproducción, Departamento de Biología y Química, Facultad de Ciencias Básicas, Universidad Católica del Maule, Talca, Chile; ^2^Escuela de Ingeniería en Biotecnología, Facultad de Ciencias Agrarias y Forestales, Universidad Católica del Maule, Talca, Chile; ^3^Centro de Investigación de Estudios Avanzados del Maule (CIEAM), Vicerrectoría de Investigación y Postgrado, Universidad Católica del Maule, Talca, Chile; ^4^Centro de Investigación en Neuropsicología y Neurociencias Cognitivas, Facultad de Ciencias de la Salud, Universidad Católica del Maule, Talca, Chile; ^5^Departamento de Biología Celular, Facultad de Ciencias Biológicas, Universidad de Concepción, Concepción, Chile; ^6^Departamento de Bioquímica y Biología Molecular, Facultad de Ciencias Biológicas, Universidad de Concepción, Concepción, Chile

**Keywords:** egg activation, polyspermy, cortical reaction, cortical granules, calcium signaling, maternal genes

## Abstract

Fertilization by multiple sperm leads to lethal chromosomal number abnormalities, failed embryo development, and miscarriage. In some vertebrate and invertebrate eggs, the so-called cortical reaction contributes to their activation and prevents polyspermy during fertilization. This process involves biogenesis, redistribution, and subsequent accumulation of cortical granules (CGs) at the female gamete cortex during oogenesis. CGs are oocyte- and egg-specific secretory vesicles whose content is discharged during fertilization to block polyspermy. Here, we summarize the molecular mechanisms controlling critical aspects of CG biology prior to and after the gametes interaction. This allows to block polyspermy and provide protection to the developing embryo. We also examine how CGs form and are spatially redistributed during oogenesis. During egg activation, CG exocytosis (CGE) and content release are triggered by increases in intracellular calcium and relies on the function of maternally-loaded proteins. We also discuss how mutations in these factors impact CG dynamics, providing unprecedented models to investigate the genetic program executing fertilization. We further explore the phylogenetic distribution of maternal proteins and signaling pathways contributing to CGE and egg activation. We conclude that many important biological questions and genotype–phenotype relationships during fertilization remain unresolved, and therefore, novel molecular players of CG biology need to be discovered. Future functional and image-based studies are expected to elucidate the identity of genetic candidates and components of the molecular machinery involved in the egg activation. This, will open new therapeutic avenues for treating infertility in humans.

## Introduction

Sexual reproduction requires the interaction of gametes, cells highly specialized for fertilization. To this end, the egg controls the male gamete entry to prevent genetic abnormalities caused by supernumerary sperm (polyspermy), which results in failed embryo development and miscarriage (Hassold et al., 1980; Evans, 2020). Polyspermy can occur in mammalian eggs by a low percentage, generally between 1 and 2% under *in vivo* conditions (Rothschild, 1954). To ensure monospermy, the female reproductive tract in mammals acts as an effective barrier or selector to the sperm. This reduces the concentration and number of viable male gametes that reach the egg (Bianchi and Wright, 2016). If polyspermy occurs, the formed zygote undergoes spontaneous abortion. Nonetheless, it has been reported that triploid and tetraploid pregnancies can progress to birth. In this case, the infants show a variety of malformations including cardiac anomalies, syndactyly (fingers or toes that are joined), hypotonia, among others (Uchida and Freeman, 1985; Sherard et al., 1986; Shiono et al., 1988; Dean et al., 1997).

Another failure of gamete interaction is the inability of either the sperm to fertilize the female gamete, or the egg to interact with the sperm, causing infertility. The World Health Organization (WHO) defines infertility as “*A disease of the male or female reproductive system defined by the failure to achieve a pregnancy after 12 months or more of regular unprotected sexual intercourse”* (WHO-ICMART revised Glossary). In humans, the infertility rate is around 15% worldwide, and near of 50% is caused by male fecundity alterations (Cui, 2010). It is well established that fertility decreases with age. Specifically, women’s fertility starts to decline over 32 years old. However, such a decrease becomes steep and critical after 37 years old (American College of Obstetricians and Gynecologists Committee on Gynecologic Practice and Practice Committee, 2014). On the other hand, male fertility starts to decline after 35 years old (Mathieu et al., 1995). In fact, several studies show that women, between 16 and 26 years old, show significantly higher pregnancy probabilities than those of 35–40 years old. Concomitantly, women’s infertility ratio increases with age: 15, 22–24, and 29% ranging in age from 19–26, 27–34, and 35–39 years old, respectively (Dunson et al., 2004). Currently, *in vitro* fertilization (IVF) is one of several alternatives to treat infertility in humans. However, the success of this technique relies on ovarian stimulation, complete oocyte maturation, concentration of sperm, and the patient’s age (van der Ven et al., 1985). Therefore, a better understanding of how fertilization is regulated may facilitate the development of diagnostic tools to assess gamete quality used in IVF practices, and its improvement.

Following sperm-egg recognition and fusion, the egg has evolved several activation mechanisms to avoid cytogenetic defects. Thus, the event of egg activation provides prevention of polyspermy, but also protection of the fertilized egg/embryo until implantation or hatching. Also, and together with fertilization, determines the transition from oogenesis to embryogenesis.

Monospermic fertilization involves the function of a primary barrier given by the female tract, the cumulus cellular layer (jelly coat or the egg jelly in marine invertebrates), an extracellular glycoprotein matrix surrounding the egg known as *zona pellucida* (ZP) in mammals (vitelline envelope in amphibians and *Drosophila*, and chorion in fish), and the egg plasma membrane (PM) (Claw and Swanson, 2012). However, in species with external fertilization, polyspermy blockade referred to as the fast and slow blocks, is critical (Rothschild and Swann, 1952). The fast or electrical block to polyspermy involves changes to the egg PM that have been well characterized in frogs and sea urchin (Jaffe and Cross, 1984), but not well understood in other animals. In most vertebrates, the slow or mechanical block to polyspermy is a key event and involves the exocytosis of cortical granules (CGs). After it is initiated, the subsequent elevation of the extracellular coat or the modification of the ZP becomes unreceptive to the sperm (Wessel et al., 2001).

**FIGURE 1:**
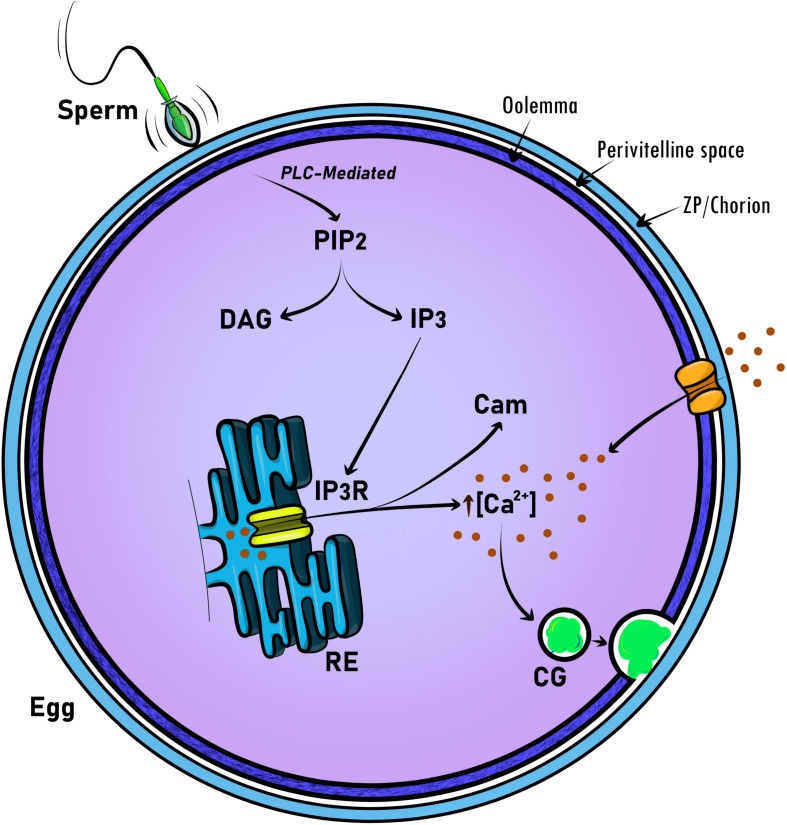
Schematic representation of universal molecular regulators acting in intracellular calcium signaling at egg activation and fertilization. To promote CG exocytosis, calcium signaling is triggered by the sperm at fertilization. Then, the generation of phosphatidylinositol 4,5-bisphosphate (PIP_2_), 1,2-diacylglycerol (DAG) and inositol 1,4,5-triphosphate (IP_3_) is mediated by phospholipase C (PLC). Finally, IP_3_ through the binding to the IP_3_ receptor (IP3R) releases Ca^2+^ from intracellular stores. Thus, calmodulin (Cam) binds Ca^2+^ to participate in egg activation progression, including meiosis resumption. In the mammalian egg, Ca^2+^-specific channels mediate the ion influx from the extracellular space and modulate Ca^2+^ oscillations. CG, cortical granule.

The release of calcium (Ca^2+^) at fertilization results in a cascade of events that includes exocytosis of CGs (Figure 1). These secretory vesicles are egg-specific membrane-bound organelles that, upon egg activation, fuse with the PM and release their content into the extracellular space. This content includes proteases, glycoproteins, and structural proteins (see Section “Modification of ZP Proteins by the CG Content” for more details) (Wessel et al., 2001). CG exocytosis (CGE) is executed after fertilization and functions to prevent polyspermy, and regulate the early embryo’s developmental progression. To facilitate this immediate response, CGs become localized at the PM during oocyte maturation. In several organisms studied, it has been proposed that the Ca^2+^ signal is transduced to control the activity of maternal determinants. These factors allow then the exocytosis of CG content to the extracellular space (Matson et al., 2006; Mei et al., 2009; Fuentes et al., 2020). Although cytosolic Ca^2+^ increases during egg activation, the factors that regulate CG biology, as well as the block to polyspermy, remain largely unknown. Here, we discuss the significant progress made in linking animal phenotypes and genetics (phenogenetics) to elucidate the molecular identity and functionality of factors regulating CGE and fertilization.

## General Mechanisms for Polyspermy Blockade

Polyspermy generates a non-diploid zygote and causes embryonic lethality in most sexual species (Rothschild, 1954; Eisen et al., 1984; Evans, 2020). Initial studies in rats and rabbits have shown that there is a time frame in which the gametes can optimally interact. Accordingly, the incidence of polyspermy sharply increases when fertilization is delayed after ovulation (Austin and Braden, 1953). Mechanical and molecular mechanisms have been described as regulators of polyspermy avoiding in different species (Wessel et al., 2001). However, the mechanisms leading to polyspermy blockade in mammals have not been completely understood (Schuel, 1978; Wong and Wessel, 2006). Currently, they are few known molecular factors regulating polyspermy prevention. These have been described in animal species such as sea urchin and frogs, as well as their function mediating fast and slow blockade (Wozniak and Carlson, 2020).

Changes in membrane potential associated with the fast blockade of the polyspermy, were initially described in echinoderms and amphibians (Jaffe and Cross, 1986). Early studies in *Strongylocentrotus purpuratus* (pacific sea urchin) indicate that this mechanism involved changes in the PM potential, leading to a depolarization that electrically prevents further fertilization from other sperm (Jaffe, 1976). This mechanism is mediated by Ca^2+^ influx, through voltage-gated Ca^2+^ channels, and Ca^2+^-dependent changes in membrane potential (Chambers and de Armendi, 1979; Swann et al., 1992; McCulloh et al., 2000). In amphibian, Ca^2+^- activated chloride channels, mediates the fast blockade of polyspermy (Cross and Elinson, 1980). These channels were recently identified as TMEM16A in *Xenopus laevis* oocytes (Wozniak et al., 2018). Given that echinoderms and amphibians are part of the deuterostome taxonomic group and are closely related to humans, it was rational to test whether the “fast polyspermy blockade” strategy is present in mammalian eggs.

Electrophysiological measurements of the PM potential in ZP-free hamster eggs show short hyperpolarization transients at fertilization. These, depending on the number of events, were associated with single-entry sperm or continuous series without any long pause when polyspermy occurs (Miyazaki and Igusa, 1981). In rabbit eggs with or without ZP, changes in the PM potential were recorded after insemination. The fertilization responses included a slow depolarization and additional “insemination potentials” were observed. Also, these are transients composed of short hyperpolarizations followed by slow depolarizations. These changes were detected only in eggs where sperms were added in the culture media, and were too slow and small to account for a PM block (McCulloh et al., 1983). Mouse eggs do not show an electrical response when fertilized (Igusa et al., 1983; Jaffe et al., 1983).

The slow or mechanical blockade of the polyspermy has been related to the cortical reaction, which includes CGE and subsequently, the extracellular coat remodeling (Fahrenkamp et al., 2020). It has been reported the presence of CG close to the PM in the cytosol of a variety of species, including sea urchin eggs (Anderson, 1968). These eggs are surrounded by an external coat known as a vitelline layer, which mediates a specific fertilization acting as a barrier to exogenous sperm from other species (Summers and Hylander, 1975).

Both CGs and the vitelline layer are critical structures participating in the polyspermy blockade. First, in sea urchins, the initial contact of the sperm with the egg triggers the formation of the “fertilization membrane,” creating an area between this membrane and the egg. This area is called perivitelline space. Second, the content of CGs is released to the perivitelline space, and just before the completion of the cortical reaction, the gametes fuse. Third, the egg forms the “fertilization cone.” Finally, and after ∼8 min of the initiation of the CG release, the hyaline layer is formed and become thicker as the fertilized egg matures. At that time, ∼14 min after insemination, the fertilization membrane is referred as chorion (Anderson, 1968).

Similar sequence of events have been observed in other invertebrate species such as starfish (Chambers, 1930; Holland, 1980; Schroeder and Stricker, 1983; Longo et al., 1995). Structurally, the extracellular coats of vertebrate eggs are composed of long, interconnected filaments that are made up of highly conserved proteins (Litscher and Wassarman, 2007). The most extensively characterized extracellular coat is the mammalian ZP, and its general characteristics have been revised in Section “Modification of ZP Proteins by the CG Content.”

As mentioned above, the exocytosis of CGs depends on the rise of intracellular Ca^2+^ ([Ca^2+^]_*i*_) (Figure 1) (see Section “Dynamics of the Ca^2+^ Levels in Animal Eggs”). The increase of ([Ca^2+^]_*i*_ as periodic oscillations, or as a single transient, is the first signal of fertilization in all species studied so far (Kashir et al., 2013). The link between the increase of [Ca^2+^]_*i*_ and CG biology has not been fully studied. In Section “Cortical Granule Exocytosis (CGE) in Eggs: Models for a Calcium-Driven Factors Release Determining Monospermic Fertilization,” we will focus on the CGE-Ca^2+^ signaling link as the main cellular association ensuring successful fertilization in animals.

Oocytes are surrounded by granulosa cells, which provide essential metabolites and molecules (Eppig, 1979). Granulosa cells extend thin processes that penetrate the ZP to reach the oocyte, called transzonal projections (TZPs). The tip of these projections exhibits a foot-like structure that increases the contact area (Macaulay et al., 2014). TZPs are actin- and microtubule-rich structures contacting the oocyte (Albertini and Rider, 1994) through gap junctions (Anderson and Albertini, 1976), and transport essential molecules participating in oocyte maturation (Eppig, 1979; Norris et al., 2009; Macaulay et al., 2016). Furthermore, the number of these structures diminishes throughout oocyte maturation by TZPs retraction (Liu et al., 2020). It has been shown that there is a relationship between TZP integrity and the perivitelline space size (Yuan et al., 2017). Notably, the perivitelline space size is related to polyspermy prevention (Yoshida and Niimura, 2011). It has been shown that TZPs are involved in polyspermy blockade since an abnormal TZP retraction allows polyspermy. Liu et al. (2020) proposed that the expantion of the perivitelline space is necessary to sever the TZP, close the pores of ZP and prevent sperm penetration on the ZP. Thus, avoiding polyspermy (Liu et al., 2020).

## Cortical Granule Biology

### CG Biosynthesis

Cortical granules were first described in sea urchin eggs 112 years ago (Harvey, 1909). In mammals, C. R. Austin was the first researcher characterizing them in hamster oocytes using phase-contrast microscopy (Austin, 1956). These secretory vesicles can be visualized as soon as the early stages of oocyte development (Gulyas, 1980). The formation of CGs in rat and hamster oocytes occurs in association with several small Golgi complexes, showing a similar morphology and size, ranging from 0.2 to 0.6 μm (Austin, 1956; Gulyas, 1980; Cherr et al., 1988). During the early stages of oogenesis, Golgi units hypertrophy and proliferate. At this stage, the formation of CGs from the Golgi complexes can be observed for the first time, migrating toward the subcortical region of the oocytes (Gulyas, 1980). From hypertrophied Golgi, small vesicles are synthesized and fused into larger ones, thus forming mature CGs that eventually separate from the Golgi complexes, and migrate to the surface or clump together in small groups (Gulyas, 1980; Liu, 2011).

**FIGURE 2:**
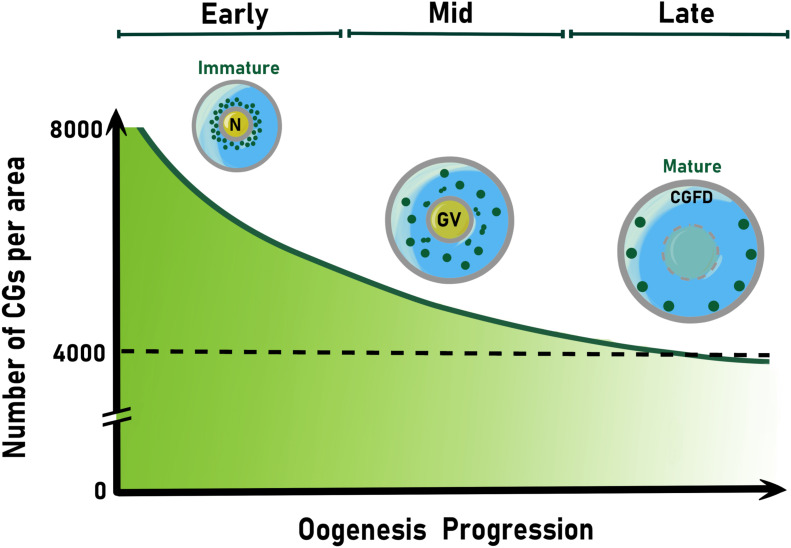
Schematic representation of cortical granule spatial localization and number reduction during oogenesis. Colored CGs (green) are shown. In the early oocyte, their biogenesis takes place and accumulate around the nucleus (N). Then, CGs move away of the germinal vesicle (GV) and translocate to oocyte periphery during mid oogenesis. In the late oocyte, CGs anchor to the cortex until their exocytosis soon after fertilization or egg activation. The null accumulation of cortical granules to one pole of the oocyte establishes the CG free domain (CGFD), present in some animal species. After CG biogenesis begins, their number decay exponentially as oogenesis proceeds. Thus, CGs number display an about 2-fold decrease at the end of oogenesis. CG, cortical granule.

In mice, the total number quantification of CGs per oocyte is higher in mature oocytes than in activated oocytes, but lower than the germinal vesicle (GV) oocytes (Figure 2). This number decreases from 8,000 to ∼4,000 CGs at Metaphase of Meiosis II (MII), when the oocyte completes its maturation (Ducibella et al., 1988b, 1990). The greater number of CGs in GV compared to mature oocytes (MII) is due to their loss through the first polar body extrusion, premature exocytosis, and biochemical modifications (Nicosia et al., 1977; Ducibella et al., 1988a). Also, by the activity of factors that have not been identified yet. However, a constant increase in peripheral CG density following oocyte maturation has been reported in mouse oocytes (Ducibella et al., 1988a, 1994). In contrast, in *in vitro* matured pig oocytes, it has been shown that the mean value of the peripheral density of CGs during mid-oogenesis was less than in early oocytes (Kulus et al., 2020). This suggests that the acquisition of meiotic competence and progression correlates with a decrease in the number of CGs per 100 μm^2^ of the ooplasmic cortex (Figure 2).

Cortical granule release has also been described in human oocytes. It was demonstrated that CG exocytosis increases in oocytes that acquired meiotic competence and their content released to the perivitelline space (Rousseau et al., 1977). Using eggs that were not fertilized during IVF procedures, Ducibella et al. (1995) demonstrated that these unfertilized eggs showed CG loss and a biochemically modified ZP (Ducibella et al., 1995). It was also shown that human eggs have two populations of CG with different diameter and density, G1 and G2. The G2 population is secreted during all stages of oocyte maturation (Hinduja et al., 1990). Additionally, CGE occurs following Intracytoplasmic sperm injection (ICSI) in fertilized and activated eggs (Ghetler et al., 1998). As shown in other species, human oocytes also undergo cytoplasmic rearrangements during maturation, including CG migration to the cortex (Trebichalska et al., 2021).

### Modification of ZP Proteins by the CG Content

The initiation of fertilization relies on the binding of the sperm to the ZP, a glycoprotein matrix that surrounds the oocyte. In mice, it was first observed in primary follicles growing during oocyte maturation (Odor and Blandau, 1969). The ZP is composed of three highly conserved proteins: ZP1 (180 kDa), ZP2 (120–140 kDa), and ZP3 (83 kDa). These factors represent 36, 47, and 17% of the mouse ZP proteins, respectively (Bleil and Wassarman, 1980a; Wassarman, 1988). Expression of ZP genes is tightly regulated by the *FIG*α gene, which has functional homologs in humans and zebrafish (Huntriss et al., 2002; Zhao et al., 2008; Wang et al., 2009; Qin et al., 2018). Mice lacking *FIG*α do not express ZP genes and are sterile (Soyal et al., 2000). A fourth ZP protein, ZP4, has been reported in humans (Lefievre et al., 2004) and rabbits (Stetson et al., 2012), but it is considered a pseudogene in mice (Spargo and Hope, 2003). Female rabbits lacking ZP4 showed a reduction in litter size, as well as a disorganized and thinner ZP (Lamas-Toranzo et al., 2019).

The ZP proteins are synthesized as precursors in the oocyte, which are then glycosylated to be secreted into the perivitelline space (Bleil and Wassarman, 1980b; Epifano et al., 1995; Boja et al., 2003). Structurally, ZP2 and ZP3 proteins consist of an N-terminal secretory signal peptide, a conserved ZP domain comprised of ∼260 amino acids, highly glycosylated since contains highly conserved cysteines, and a C-terminal propeptide with a single-spanning transmembrane domain (Bork and Sander, 1992). The extracellular coat is referred to as the chorion or vitelline envelope in fish and amphibians, respectively. Phylogenetically, the extracellular coat proteins of these species are similar to those in mammals, suggesting high evolutionary conservation (Monne et al., 2006). Although it has been shown that polyspermy prevention relies mostly on CGE, there are still additional mechanisms that needs to be better investigated (i.e., electrical polyspermy blockade in mammals, specific recognition between the sperm and egg that can certainly affect the polyspermy blockade, among others). Additionally, less is known about how the contents of CGs modify the extracellular coat and interact with ZP-proteins to ensure a definitive sperm blocking.

**FIGURE 3:**
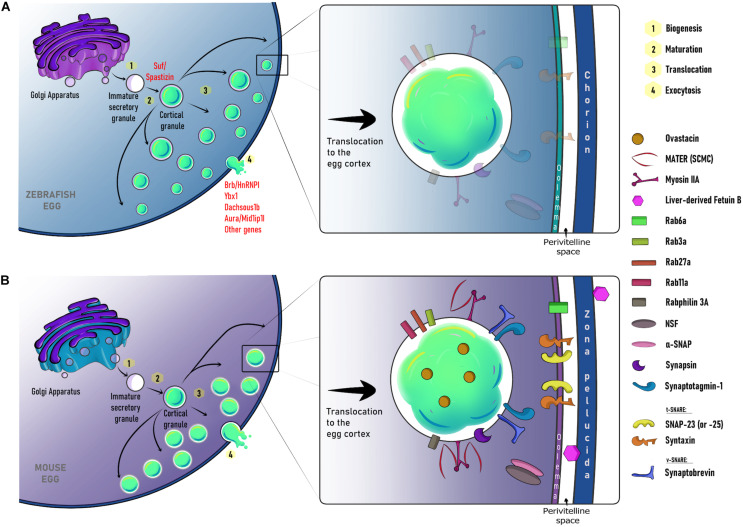
Schematic representation of major steps and molecular regulators for cortical granule biogenesis, translocation, and exocytosis in the zebrafish **(A)** and mouse **(B)**. In the early oocyte, CGs are formed from the Golgi as immature secretory vesicles. In zebrafish, their maturation is regulated by Suf/Spastizin. During mid and late oogenesis, mature CGs are recruited and translocated to the oocyte cortex. This process is coordinated by an actin-based network and several maternal factors including Rab27a and Rab11a. Ultimately, MATER functions to anchor CGs at the cell cortex. By egg activation/fertilization, their content is exocytosed. This process is regulated by maternally-loaded molecules such as Brb/HnRNP I, Ybx1, Dachsous 1b, Aura/Mid1p1l in the zebrafish egg, and Rab3a, Rabphilin 3A, Rab6a, and the SNARE complex in the mouse egg. Notice that shaded proteins represent those maternally-loaded molecules that could also be present in the zebrafish female gamete. CG, cortical granule; SCMC, subcortical maternal complex.

The content of CGs in mammals is estimated to be ∼100–350 picograms of proteins (Green, 1997). Several studies have demonstrated the presence of glycosylated components, proteinases, ovoperoxidase, calreticulin, *N*-acetylglucosaminidase, p32, and peptidylarginine deiminase (Hoodbhoy and Talbot, 1994; Liu, 2011). The release of the CG content promotes the modification of the ZP by chemically modifying its proteinaceous components. A key component of the CG content is ovastacin (Figure 3), an oocyte-specific zinc metalloendopeptidase encoded by the mouse *Astl* gene (Burkart et al., 2012). This protein is initially stored in the CGs and then exocytosed to the perivitelline space after fertilization. The target glycoprotein of ovastacin function is the ZP2, particularly the domain ZP2^51–149^, which is essential for both gamete binding and female fertility (Avella et al., 2014). Once the sperm binds to ZP2, it triggers the acrosome reaction. The migration of CGs to the cortex is critical for posterior CGE. Next, the gametes fuse, and the CGs exocytose their content (Wessel et al., 2001; Ducibella et al., 2002; Wong and Wessel, 2006; Vogt et al., 2019). Ovastacin cleaves ZP2 and mutants lacking its activity show that the sperm binds to the ZP even in a 2-cell stage embryo (Hinsch and Hinsch, 1999; Gahlay et al., 2010; Burkart et al., 2012). These results highlight the importance of CG-derived factors for successful ZP remodeling and monospermic fertilization.

### CG Transport and Cortex Accumulation

In mammals, CGs are constantly formed during early oogenesis, but their spatial localization changes as meiosis progresses (Liu, 2011). In zebrafish, CG localization at the PM is thought to be due to displacement by yolk proteins accumulating in the center of the oocyte (Selman et al., 1993; Fernandez et al., 2006). In frog and mouse, CG localization depends on their transport along actin filaments, but independent of the microtubule cytoskeleton, as occurs in mammals and sea urchin oocytes (Wessel et al., 2001, 2002).

In pigs, CGs are distributed in the center of the early oocyte. Yet, during mid-oogenesis, a concentration twice higher of these secretory vesicles can be found at the cell periphery, suggesting their translocation from the central region toward the cortex. Moreover, CG translocation to the cortical area is associated with a high meiotic competence (Kulus et al., 2020). By analyzing fixed mouse oocytes, it has been shown that CG transport to the PM is a microfilament-dependent process (Connors et al., 1998; Wessel et al., 2002). In both mouse and sea urchin oocytes, it has been suggested that CGs bind to the actin cytoskeleton at the beginning of meiotic maturation. Then, they migrate through the oocyte without a microtubule-based contribution (Wessel et al., 2002; Cheeseman et al., 2016). In this context, CGs move along a cytoplasmic actin network in a process regulated by Rab27a, whose function allows their translocation to the PM (Figure 3). Additionally, Rab27a mutants have shown an increase in polyspermy due to a total absence of CG recruitment to the oocyte cortex (Cheeseman et al., 2016). It has also been shown that CG transport to the PM is controlled by Rab11a. It transiently binds to CGs and increases their translocation speed toward the cortex in a myosin Vb-dependent manner (Schuh, 2011; Cheeseman et al., 2016). On the other hand, the CG anchor in the egg cortex has been associated with the maternal gene MATER (Figure 3). Thus, MATER, located at the subcortical maternal complex (SCMC), determines their docking at the egg cortex and controls the cortical actin clearance promoting CGE (Vogt et al., 2019).

The distribution of CGs at the egg cortex varies between species. For example, areas where CGs are not present (CG free domain, CGFD) have been described in hamster (Szollosi, 1967; Okada et al., 1986), mouse (Ducibella et al., 1990), and rat oocytes (Szollosi, 1967) (Figure 2). In mice, the formation of distinct CGFD was associated with metaphase I and metaphase II chromosomes (Connors et al., 1998). Apparently, this region was first described as exclusive in rodents, since oocytes from felines, equines, bovines, pigs, and humans lack this domain (Liu, 2011). However, zebrafish eggs also lack CGs at the so-called animal pole, which gives rise to the developing embryo (Nelsen, 1953). Rab6a is one of the proteins functioning in CGFD formation. In fact, Rab6a knock-down mice exhibit a 50% reduction of CGFD formation (Ma et al., 2016). This indicates that the formation of a well-defined CG-free cytoplasmic domain is a conserved and Rab proteins-mediated mechanism of oocyte behavior for fertilization preparation. It has been hypothesized that the function of CGFD is to protect the maternal chromatin. This hypothesis is supported by the fact that sperm-egg fusion occurs in a low frequency in this area (Johnson et al., 1975). However, the CGFD physiological significance and its function at fertilization remain elusive.

## Cortical Granule Exocytosis (CGE) in Eggs: Models for a Calcium-Driven Factors Release Determining Monospermic Fertilization

### Overview of the Role of Ca^2+^ Signaling in Egg Activation

In sexual reproductive organisms, the fusion of female and male gametes is a critical step to trigger a series of events that will lead to embryo development. These occur during a developmental frame window known as egg activation, and are characterized by several sequential steps, including two main events: CGE and cytoplasmic reorganization (Fuentes et al., 2018, 2020; Wakai et al., 2019). The egg, in response to fertilization, displays several transient elevations of [Ca^2+^]_*i*_, known as calcium oscillations, which promote CGE and cytoplasmic movements. Calcium oscillations are accompanied by synthesis and posttranslational modifications of new proteins (Potireddy et al., 2006; Roux et al., 2006), maternal mRNAs degradation, *de novo* transcription of zygotic RNAs (Hamatani et al., 2004), among other key events necessaries for early pre-implantation development [for review (Krauchunas and Wolfner, 2013)]. Moreover, the number of sperm-heads that fuse at fertilization determines the frequency of Ca^2+^ oscillations, increasing with the number of sperms fertilizing. Thus, in polyspermic eggs Ca^2+^ starts oscillating earlier than in monospermic ones (Faure et al., 1999).

It is well-established that a rise in [Ca^2+^]_*i*_ is universally required for egg activation. Thus, in all animals, it appears that the rise in [Ca^2+^]_*i*_ release involves activation of the phosphoinositide (PI) pathway, which results in the production of inositol 1,4,5-triphosphate (IP_3_) and 1,2-diacylglycerol (DAG) (Figure 1). IP_3_ then binds to its receptor (IP_3_R) on the ER, promoting the release of Ca^2+^ (Miyazaki et al., 1992; Xu et al., 1994; Sharma and Kinsey, 2008). An important downstream effector of this Ca^2+^ signaling is Ca^2+^/calmodulin-dependent kinase II (CaMKII), which also controls egg activation in most of the organisms studied up to date (Markoulaki et al., 2003; Knott et al., 2006; Backs et al., 2010).

Genetically, the study of Ca^2+^ signaling and its role in egg activation has revealed that CGE relies on maternally inherited molecules [reviewed in Fuentes et al. (2018)]. However, the identity of most of the Ca^2+^-dependent regulators and mechanisms orchestrating egg activation in animals remains unknown. We will discuss current knowledge of maternal factors implicated in CGE in Section “Genetic Regulation of CG Biology During the Oocyte-to-Embryo Transition: Lessons From Mouse and Zebrafish Model Systems.”

### Dynamics of the Ca^2+^ Levels in Animal Eggs

In sea urchin and *Xenopus*, fertilization triggers several key events that promote the egg-to-embryo transition. The sperm contribution does not consist of DNA only, since it also delivers phospholipase Cγ (PLCγ) to the haploid egg (Lee and Shen, 1998; Carroll et al., 1999; McDougall et al., 2000; Sato et al., 2006; Bates et al., 2014). This enzyme converts the phosphatidylinositol 4,5-bisphosphate (PIP_2_) present in the PM into IP_3_ (Ciapa et al., 1992; Snow et al., 1996). The rise of IP_3_ concentration induces Ca^2+^ release from the ER through the activation of IP_3_ receptor (IP_3_R); thus, increasing the [Ca^2+^]_*i*_ (Steinhardt et al., 1977; Kubota et al., 1987; Runft et al., 1999). Microinjection of heparin, an IP_3_R antagonist, decreases the rate of increase in [Ca^2+^]_*i*_ and the propagation speed throughout the fertilized egg in sea urchin (McDougall et al., 2000). Ryanodine receptor (RyR), a Ca^2+^ channel sensitive to Ca^2+^ ions and caffeine, is also involved in its release from the ER during fertilization in these eggs. Ruthenium red, another and unspecific RyR antagonist does not entirely block Ca^2+^ release from the ER. However, when ruthenium red and heparin are co-administered into sea urchin eggs, the increase of Ca^2+^ is completely blocked (Galione et al., 1993). In sea urchin, the Ca^2+^ wave initiates and propagates throughout the cell in ∼20–30 s after the sperm-egg interaction (McDougall et al., 2000). The elevation of [Ca^2+^]_*i*_ consists of a single peak that propagates across the cell and lasts for ∼2–3 min (Steinhardt et al., 1977). This increase in the [Ca^2+^]_*i*_ is sufficient to resume the cell cycle (Steinhardt and Epel, 1974). This signaling mechanism is also conserved in *X. laevis* eggs (Larabell and Nuccitelli, 1992; Nuccitelli et al., 1993; Runft et al., 1999).

The increase of [Ca^2+^]_*i*_ in response to fertilization in *Xenopus* eggs involves Ca^2+^ release from the ER mediated by the activation of the IP_3_R. Furthermore, in this vertebrate organism, microinjection of IP_3_ is sufficient to induce the Ca^2+^ waves throughout the egg cortex (Busa et al., 1985). The Ca^2+^ wave in *X. laevis* is not homogeneously distributed throughout the egg, showing lower Ca^2+^ concentration in the cytoplasm compared to the cortex. Moreover, the speed Ca^2+^ propagation also varies. Thus, the cortical wave travels faster (8.9 μm/s) than the cytoplasmic (5.7 μm/s) Ca^2+^ wave to the center of the egg (Fontanilla and Nuccitelli, 1998). In addition to the Ca^2+^ dynamics, a protein kinase C (PKC) wave has also been detected during *Xenopus* egg activation (Larabell et al., 2004). This wave is triggered by the production of DAG as a product of PIP_2_ hydrolysis. DAG activates PKC, producing a wave that follows the Ca^2+^ one, which propagates at the same speed and it is critical to CGE (Larabell et al., 2004).

In zebrafish, the released Ca^2+^ is necessary and sufficient for many egg activation events, including CGE (Mei et al., 2009; Fuentes et al., 2018). There are two regionalized Ca^2+^ waves that originate at the point of sperm-egg interaction, or animal pole, and culminate at the vegetal pole (Sharma and Kinsey, 2008). Also, the formation of well-defined cytoplasmic domains during oogenesis spatially restricts signaling components such as Src family kinases (SFKs) and PLCγ. These molecules participate in providing the molecular basis of egg activation in this species (Sharma and Kinsey, 2006; Fuentes et al., 2018). Ultimately, the study of genetic models (i.e., maternal-effect mutants; see Section “Insights Into the Maternally Regulated Mechanism of ZP and Perivitelline Space Formation”) has revealed egg components regulating its activation, Ca^2+^ dynamics, and CG biology (Mei et al., 2009; Kanagaraj et al., 2014; Li-Villarreal et al., 2015; Eno et al., 2016).

Monitoring of Ca^2+^ in real-time with luminescence and either fluorescent probes or tagged associated proteins has been a valuable approach for the study of egg activation *in vivo*. These tools have revealed how [Ca^2+^]_*i*_ behaves in a given developmental period during early embryogenesis (Miyazaki et al., 1986; Whitaker, 2006; Sharma and Kinsey, 2008). [Ca^2+^]_*i*_ waves can last over as short as seconds, or more sustained signals as hours (Kashir et al., 2013). Spatially, these can either be visualized cortically or invading the central region of the zebrafish egg (Sharma and Kinsey, 2008). Such behavior might be possible thanks to internal Ca^2+^ release, mainly from the ER (Mei et al., 2009; Machaty et al., 2017), although it is known that the maintenance of the Ca^2+^ oscillations in mouse oocytes is depending on extracellular Ca^2+^ (Kline and Kline, 1992).

Finally, in mammals, [Ca^2+^]_*i*_ oscillations are fundamental for three critical processes taking place during egg activation. First, initial Ca^2+^ oscillations are responsible for the resumption of the cell cycle. Specifically, the egg finishes meiosis II, inhibiting the Maturation or M-Phase promoting factor (MPF) through a CamK II process. MPF is a protein complex form by the cyclin B-Cdk1 dimer (Arion et al., 1988; Dunphy et al., 1988; Gautier et al., 1988; Draetta et al., 1989; Labbe et al., 1989; Meijer et al., 1989; Gautier et al., 1990) and great wall kinase (Gwl) (Hara et al., 2012). Second, Ca^2+^ oscillations are involved in the pronucleus formation by reducing the activity of mitogen-activated protein kinase (MAPK). After the pronucleus formation during the first interphase, Ca^2+^ oscillations cease. Third, Ca^2+^ oscillations induce the release of CG. The CGE is finished between the first hour after sperm-egg fusion (Stewart-Savage and Bavister, 1991; Tahara et al., 1996).

During the fusion of gametes, mammalian sperm releases PLCζ (Saunders et al., 2002; Kouchi et al., 2004). This protein hydrolyzes phosphatidylinositol 4,5-biphosphate (PIP_2_) to IP_3_ and DAG. The incorporation of PLCζ into the oocyte triggers the production of IP_3_ and the release of Ca^2+^ from the ER through the activation of the IP_3_R (Miyazaki et al., 1993; Saunders et al., 2002; Kurokawa et al., 2004) (Figure 1). Thus, sperms displaying a down-regulated PLCζ expression exhibit a reduction or an absence of Ca^2+^ oscillations (Knott et al., 2005). Interestingly, mice lacking PLCζ produces sperms that are not able to trigger Ca^2+^ oscillations, showed severely reduced fertility. However, they are not completely infertile, suggesting an additional mechanism(s) to ensure fertility. Fertilized eggs with null-PLCζ showed a higher rate of polyspermy, confirming the role of Ca^2+^ oscillations in monospermic fertilization (Hachem et al., 2017; Nozawa et al., 2018). Ca^2+^ oscillations last for hours (Cuthbertson and Cobbold, 1985; Miyazaki et al., 1986); however, to be maintained, Ca^2+^ influx from the extracellular media is needed (Kline and Kline, 1992; Wakai et al., 2011). Ca^2+^-permeable channels are expressed in the oocyte during the maturation process, and at the MII stage (Carvacho et al., 2018). These channels are responsible for replenishing the intracellular ER stores during oocyte maturation and would contribute to the Ca^2+^ influx during egg activation (Miao et al., 2012; Carvacho et al., 2013, 2016, 2018; Bernhardt et al., 2018) (Figure 1).

### Dynamics of Ca^2+^-Dependent Proteins During CGE in Animal Eggs

Spatiotemporal organization of maternally-inherited molecules and Ca^2+^-dependent protein functions are fundamental to orchestrate egg activation. Several mutant and knock-down animals, displaying abnormal phenotypes during the oocyte and egg development, have flourished our knowledge of the factors regulating this process (Mei et al., 2009; Kanagaraj et al., 2014; de Paola et al., 2015; Li-Villarreal et al., 2015; Cheeseman et al., 2016; Eno et al., 2016; Vogt et al., 2019). Also, as spatially restricted molecular profiles are also perturbed in maternal-effect mutants, it is possible to dissect functional relevance and genotype–phenotype associations during the oocyte-to-egg transition, including those associated with CG behavior (Figure 3). To study them, a combination of imaging and pharmacological tools has been pivotal to decipher Ca^2+^-dependent mechanisms during egg activation (Mei et al., 2009; Kanagaraj et al., 2014; Li-Villarreal et al., 2015; Eno et al., 2016). These approaches are expediting the description of cellular, molecular, and physiological phenotypes during CG biology in animal species.

In sea urchin eggs, CGE is controlled by a protein complex sensitive to Ca^2+^ called SNARE (Soluble *N*-ethylmaleimide sensitive-factor attachment protein receptor). This complex is composed of proteins attached to the membrane enclosing the secretory vesicle (vSNARE), and present in the target membrane such as the PM (tSNARE). To allow a rapid release of the CG content after fertilization, these vesicles are docked to the PM through the interaction of vSNARE with tSNARE proteins. The vSNARE protein expressed in sea urchin eggs is synaptobrevin (also called VAMP) (Avery et al., 1997). The tSNARE proteins expressed in these eggs are syntaxin and SNAP-25 (Avery et al., 1997; Conner et al., 1997; Coorssen et al., 2002).

Another critical protein for CGE present into CGs is synaptotagmin-1 (syt1) (also known as p65) (Leguia et al., 2006). Syt1 contains two motifs that are sensitive to Ca^2+^ ions, C2A and C2B, that are important for exocytosis (Perin et al., 1990, 1991). Also, synaptobrevin, syntaxin, and SNAP-25 interact to form a molecular zipper allowing CG docking to the PM (Gao et al., 2012; Yoon and Munson, 2018). This conformation forms a *trans*-SNARE complex and when [Ca^2+^]_*i*_ increases, the ions bind to the C2 domain of syt1. This binding favors the association of this protein to the SNARE complex (Leguia et al., 2006). This interaction also induces conformational changes that trigger the zippering of the SNARE complex; thus, promoting the fusion of CGs with the PM (Gao et al., 2012). In addition, the sec1 and munc18 proteins (also known as SM proteins) also regulate CG fusion with the PM through the binding to syntaxin (Leguia and Wessel, 2004). This molecular contact stabilizes syntaxin in the SNARE complex (Dulubova et al., 1999).

On the other hand, *Xenopus* eggs exocytose CGs in a Ca^2+^-independent manner to block polyspermy. In fact, the activation of the isoform η of the protein kinase C (PKCη) is crucial to initiate CGE (Bement and Capco, 1989; Gundersen et al., 2002). Thus, the inhibition of PKCη by retinoid acid blocks egg activation in frogs. In addition, it has been shown that myosin 1e is expressed in *Xenopus* oocytes and eggs, and upon CGE stimulation, it relocalizes and associates with the vesicles. Functionally, disruption of this motor protein inhibits CGE (Schietroma et al., 2007).

In zebrafish, the spatial and temporal localization of maternally-deposited Ca^2+^ effectors within the egg would be critical for its activation progression (Mei et al., 2009; Kanagaraj et al., 2014; Li-Villarreal et al., 2015; Eno et al., 2016). These factors are spatially restricted and functioning into cortical and central cytoplasmic domains (Fuentes et al., 2018; Fuentes et al., 2020) (Figure 3A). As in mammals, a cortical Ca^2+^ wave triggers the exocytosis of CGs in zebrafish, while the central wave promotes the actin-dependent reorganization of the cytoplasm (Fuentes and Fernandez, 2010; Ajduk et al., 2011; Fuentes et al., 2018; Shamipour et al., 2019). Additionally, the organization and function of the actin cytoskeleton has also been studied in the zebrafish egg, where it plays a critical role in CGE (Becker and Hart, 1999; Mei et al., 2009). Whether an actin network participates in CG translocation during zebrafish oogenesis has to be demonstrated.

In mammalian MII eggs, one of the tSNARE proteins expressed is syntaxin 4, which is localized in the PM together with the CGs (Iwahashi et al., 2003). Nonetheless, its participation in CGE has not been shown. Ikebuchi et al. (1998) showed that SNAP-25 critically functions in CGE in mouse eggs, since its cleavage by botulinum neurotoxin A blocks this process (Ikebuchi et al., 1998). In contrast, Mehlmann et al. (2019) showed that SNAP-23, but not SNAP-25, is expressed in mouse MII eggs (Mehlmann et al., 2019). This group also found that SNAP-23 plays a role in CGE at the PM. In addition, incubation of eggs with a specific antibody against this protein inhibits its function and prevents CGE (Mehlmann et al., 2019). The unspecificity of the antibodies used by Ikebuchi et al. (1998) could explain the discrepancy between these studies (Ikebuchi et al., 1998) (Figure 3B).

VAMP is also expressed in GV and MII mouse oocytes and eggs. Particularly, VAMP1 and VAMP3 mRNAs are present and translated into proteins in MII eggs. Both isoforms, but not VAMP2, are critical for CGE since microinjection of the light chain of tetanus toxin or anti-VAMP1 and anti-VAMP3 antibodies impairs this process (de Paola et al., 2021) (Figure 3B).

The protein complex that determines CG docking in the egg cortex senses the increase in the [Ca^2+^]_*i*_ that triggers their exocytosis (Zhu et al., 2019). However, most of the molecular aspects of the signal transduction regulating this process remain to be discovered. Mammalian syt1 plays an important role in the exocytosis pathway. Syt1 has been described as associated with synaptic vesicles of neurons (Geppert et al., 1994) and chromaffin granules (Schonn et al., 2008). As in sea urchin eggs, syt1 also interacts with SNARE proteins; thus allowing the fusion of the CG membrane with the egg’s PM. In addition, it has been shown that knocking down Syt1 in mice results in both the inhibition of [Ca^2+^]_*i*_ and CGE impairment (Zhu et al., 2019) (Figure 3B).

Another important factor regulating CG biology is the GTPase Rab3A. This GTPase colocalizes with CG in mouse oocytes and is not expressed peripherally after their exocytosis. The injection of an antibody against Rab3A blocks CGE in a concentration-dependent manner, indicating a critical role of this factor in this process (Bello et al., 2016). Rabphilin-3A, a Rab3A interactor partner, is also expressed in mouse oocytes. Rabphilin-3A has within its structural features, C2 domains homologous to the synaptotagmin C2 domains. These domains specifically bind Ca^2+^ (Shirataki et al., 1993), as well as Rab3A-GTP, alpha-actinin, and ß-adducin (Yamaguchi et al., 1993; Miyazaki et al., 1994; Kato et al., 1996). It has also been shown that Raphilin-3A spatially localizes at the cortical region of the oocyte and is involved in Ca^2+^-dependent CGE. This is believed since the injection of either the N- or C-terminal region of Rabphilin-3A into mouse oocytes inhibits the exocytosis pathway (Masumoto et al., 1996) (Figure 3B). Rab3a is also expressed in sea urchin eggs (Avery et al., 1997). In fact, microinjection of the effector peptide of this factor into these cells prevent CGE. Co-microinjection of the effector peptide with IP_3_ also blocked CGE, suggesting that Rab3a functions after the docking of the CGs to the PM (Conner and Wessel, 1998). Once CGs undergo exocytosis and release their content, the complex turns into a *cis*-SNARE configuration (Stein et al., 2009).

It has been shown that α-SNAP, γ-SNAP, and NSF are expressed in mouse GV and MII oocytes. However, only α-SNAP and NSF are essential for CGE since the microinjection of either antibodies against them or their mouse mutant versions, impairs this process (de Paola et al., 2015). Whether other factors regulating SNARE function participate in CGE is still unknown, and remains to be explored.

## Genetic Regulation of CG Biology During the Oocyte-To-Embryo Transition: Lessons From Mouse and Zebrafish Model Systems

### Overview of the Maternally Controlled Egg Activation

One of the first steps in animal development is the transition from the oocyte to a developmentally active and totipotent early embryo –the oocyte to embryo transition (Whitaker, 2006). This critical developmental window relies on the expression of the maternal genome in the oocyte and the function of its gene products in the developing embryo. Thus, maternally-provided factors contribute to executing dramatic changes at the molecular level in the fertilized/activated egg and zygote, and by doing so, it conducts essential activities by the early embryo. These include the correct regulation of the cell cycle, synchronic cleavage divisions, axis patterning, and ultimately, dramatic changes in the zygotic genome structure organization (Fuentes et al., 2020).

The oocyte is a highly differentiated and transcriptional silent cell type and, prior to the initiation of maturation, it is arrested at the prophase of meiosis I. Following release from arrest, the oocyte resumes meiosis I and begins meiosis II. Then it is arrested again at metaphase II until fertilization. With the multitude of functions that regulate mRNAs and proteins prior to gametes interaction to form a zygote, the maternal genetic program also triggers the initiation of complex and spatially distributed cellular responses in preparation for egg activation (Clift and Schuh, 2013).

The egg activation events, such as cortical reaction and cytoplasmic reorganization, are largely driven by the function of maternal gene products. These functions underlie the importance of the exact timing and the amount of their production during oogenesis and prior fertilization to control this process (Fuentes et al., 2020). However, knowledge of the molecular identity of most of these maternal factors remains incomplete. Also, the post-translational regulation, action, and functional significance of these maternal factors in determining egg activation and embryogenesis progression are poorly understood.

In this section, we discuss why the zebrafish and mouse emerge as phenogenetic model systems to study maternal gene function during the oocyte-to-embryo transition. Additionally, maternal-effect mutants represent a unique tool to understand how fundamental aspects of egg activation are regulated and coordinated. This maternal control of egg activation is critical for the onset of zygote formation and proper embryogenesis. Further gene discovery by using these tools will be pivotal to understand the evolutionary conservation of the mechanisms governing egg activation. It will also provide new insights into egg activation failures, and help to understand human infertility from a molecular and phenotypic perspective.

### Insights Into the Maternally Regulated Mechanism of ZP and Perivitelline Space Formation

As we discussed earlier, the ovulated mammalian egg is surrounded by an extracellular coat called the ZP, known as the chorion in fish species. For the egg to be fertilized, sperm first penetrate the corona radiata (or granulosa cells). Then, it binds to the ZP (Avella et al., 2014) and is specifically recognized by the egg receptor Juno (Bianchi et al., 2014). The acrosomal reaction allows the sperm to penetrate the cumular cells and the ZP, and finally, fuse with the egg’s PM (Kim et al., 2008).

In most teleost species, such as zebrafish, sperm lack an acrosome and enter the egg through a funnel-shaped structure called the micropyle (Hart and Yu, 1980; Wong and Wessel, 2006; Yanagimachi et al., 2017). As a result, the sperm does not need to bind directly to the ZP, and it is proposed that ZP proteins in zebrafish are purely structural (Wang and Gong, 1999; Onichtchouk et al., 2003; Aagaard et al., 2006). The study of maternal-effect mutants suggests that gene expression products modulate the separation of the chorion from the egg’s PM to form the perivitelline space or chamber (Mei et al., 2009; Kanagaraj et al., 2014; Eno et al., 2016; Hau et al., 2020). These molecules are supplied by the mother during oogenesis. Genetic screens in zebrafish have identified a small set of mutant genes acting in CG biology (Mei et al., 2009; Kanagaraj et al., 2014; Li-Villarreal et al., 2015; Eno et al., 2016; Sun et al., 2018). For instance, maternal-effect *brom bones* (*brb*)/*heterogeneous nuclear ribonucleoprotein I* (*hnRNP I*) mutants are ventralized, and display CGE and a chorion elevation defect. It indicates that this gene is required for egg activation (Mei et al., 2009). Remarkably, activated mutant eggs have disrupted the ER IP_3_-dependent Ca^2+^ release. Also, increases in [Ca^2+^]_*i*_ at fertilization are required for CGE and actin remodeling. These findings suggest an additional role for *brb*/*hnRNP I*, as a regulator of the actin cytoskeleton-based kinetics of CGE in a Ca^2+^-dependent manner (Mei et al., 2009). The association between actin filaments function and CGE pathway has also been demonstrated by studying additional mutant phenotypes such as *dachsous1b* and *aura*/*mid1ip1l* (Figure 3A) (Li-Villarreal et al., 2015; Eno et al., 2016).

Another maternal factor, Souffle (Suf), is also required for controlling CG function during egg activation (Kanagaraj et al., 2014). In *suf* mutant oocytes, CGs are smaller and do not exocytose in the egg. Interestingly, mutant eggs also display defects in the perivitelline space formation and remodeling of the egg surface, likely due to alterations of the rate of actin polymerization (Kanagaraj et al., 2014). The mutant gene encodes the Spastizin protein, which modulates secretory granule maturation and it is implicated in Hereditary Spastic Paraplegia disease in humans (Hanein et al., 2008; Slabicki et al., 2010; Hirst et al., 2013). Therefore, Suf/Spastizin functions to form CGs from immature secretory granules and to control their fusogenic activity during oogenesis and egg activation, respectively (Figure 3A) (Kanagaraj et al., 2014). Recently, it was found that maternal *ybx1* crispant oocytes fail to mature. In addition, mutant eggs display severe CG accumulation, thus exhibiting a penetrant chorion elevation defect phenotype (Sun et al., 2018). These findings have revealed a new factor acting in vertebrate oocyte maturation and egg activation. Ybx1 regulates protein translation, therefore, further analyses of the maternal-effect *ybx1* mutant would shed light into the translational state of proteins orchestrating CGE and chorion elevation after fertilization (Figure 3A).

In mammals, the ZP is important for species-specific sperm-egg binding (Bianchi and Wright, 2020). A low percentage of immature oocytes from ZP1-null mice, show ectopic granulosa cells in the perivitelline space, which is accentuated in MII eggs likely due to the lack of ZP integrity. Interestingly, female mice showed only decreased fertility (Rankin et al., 1999). The ZP of ZP2-null mice is thinner compared to a wild-type egg, since it fails to form and stabilize the matrix generating a complete lack of the ZP. The females are infertile and do not produce early embryos and live birth mice (Rankin et al., 2001). Additionally, it has been demonstrated that ZP2 mediates sperm binding to the egg (Avella et al., 2014). This protein is the direct substrate of ovastacin factor that cleaves the N-termini domain of ZP2 (Gahlay et al., 2010; Burkart et al., 2012; Avella et al., 2014; Tokuhiro and Dean, 2018). Intriguingly, ovastacin-null mice (*Astl*^*null*^) are subfertile, suggesting still unknown additional mechanisms regulating polyspermy blockade in mammals.

Mice lacking the *Zp3* gene do not show a zona matrix and are sterile (Liu et al., 1996; Rankin et al., 1996). ZP3-null females can ovulate a low percentage of eggs without ZP, but early embryos do not develop (Rankin et al., 1996). Loss-of-function experiments show that ZP2 and ZP3 are necessary molecules for ZP and the perivitelline space formation, allowing normal fertilization and early embryo development (Liu et al., 1996; Rankin et al., 1996).

ZP proteins are heavily glycosylated (Bork and Sander, 1992). Initially, it was suggested that sperm attaches to the ZP3 through *O*-glycosylation sites (Florman and Wassarman, 1985). However, it was shown that O-glycans are not required for neither sperm binding nor fertilization (Williams et al., 2007). In fact, using ZP mutants, it was shown that CGE, modification of the ZP2 and polyspermy prevention are glycan-independent gamete recognition processes (Tokuhiro and Dean, 2018).

On the other hand, *N*-glycosylation emerges as a critical post-translational modification for embryo development (Yonezawa et al., 1995; Nakano et al., 1996; Shi et al., 2004). To date, there is only one known regulator of *N*-glycosylation during vertebrate oogenesis, the Mgat1 factor (Shi et al., 2004). It functions in the medial Golgi to modify the target polypeptide chain by adding glycans (Kumar et al., 1990). When Mgat1 is deleted in mammalian ovaries, it causes a lack of complex or hybrid *N*-glycans, thinner ZP, and reduced perivitelline space. Furthermore, female mutants have decreased fertility and a percentage of the embryos showed a retarded embryonic development (Shi et al., 2004). These altered phenotypes suggest that *N*-linked glycosylation acts as a regulatory mechanism during oogenesis. Whether Mgat1-mediated post-translational regulation controls CG biology and the function of associated factors during the oocyte-to-embryo transition, remains still unresolved.

As we mentioned earlier, during meiotic maturation and prior to fertilization, there is an early release of a small number of CGs (Nicosia et al., 1977; Ducibella et al., 1988a). The ovastacin-mediated premature cleavage of ZP2 hardens the ZP and prevents sperm binding to the egg (Burkart et al., 2012). Nonetheless, this proteolytic activity is inhibited by micromolar concentrations of the liver-derived plasma protein Fetuin-b. This highly specific inhibitor of ovastacin prevents the ZP hardening before fertilization (Dietzel et al., 2013; Korschgen et al., 2017; Karmilin et al., 2019). Fetuin-b is a member of the cystatin superfamily encoded by the FETUB gene in humans and mice, sharing 61% homology (Olivier et al., 2000). Fetuin-b is produced by the liver and is secreted to the peripheral tissues through the circulatory system (Denecke et al., 2003). Fetuin-b-null mice are infertile due to the premature cleavage of ZP2 (Dietzel et al., 2013). Thus, when fertilization occurs, CGs release a large amount of ovastacin that overcomes inhibition by fetuin-b and promotes the ZP hardening, and subsequent polyspermy blockade (Dietzel et al., 2013; Stocker et al., 2014) (Figure 3B).

### Insights Into the Maternally Regulated Mechanism of Ca^2+^-Influx and CGE

Several Ca^2+^-permeable channels are expressed in mouse eggs. One of them is the voltage-gated Ca^2+^ channel 3.2 (Cav3.2) that belongs to the T-type family (Ramirez et al., 2017). It exhibits low-threshold voltage activation and it has been shown to be expressed in the mouse egg (Peres, 1987; Bernhardt et al., 2015). Cav3.2 channels contribute to the accumulation of Ca^2+^ in the ER during maturation (Bernhardt et al., 2015). Alternatively, the TRPV3 channel, a Ca^2+^ channel that belongs to the vanilloid subfamily of the Transient Receptor Potential (TRP) channel family, is differentially expressed during mouse oocyte maturation, reaching its higher PM expression prior to fertilization (Carvacho et al., 2013; Lee et al., 2016). The activation of TRPV3 can trigger massive Ca^2+^ influx leading to parthenogenetic activation (Carvacho et al., 2013). Additionally, TRPM7, a TRP channel that belongs to the melastatin subfamily, has been identified in mouse oocytes, eggs, and also in 2-cell stage embryos (Carvacho et al., 2016). TRPM7 activity can be regulated by voltage, pH, magnesium, spermine (Kozak et al., 2002, 2005), and PIP_2_ (Runnels et al., 2002). In addition, it has been shown that TRPM7 promotes Ca^2+^ influx, contributing to replenishing the ER stores and modulating Ca^2+^ oscillations during fertilization (Bernhardt et al., 2018). Finally, Cav3.2 and TRPV3 double KO have shown decreased fertility, altered oocyte ER Ca^2+^ dynamics (fill and re-fill), and severely impaired Ca^2+^ oscillations in response to fertilization (Mehregan et al., 2021).

As discussed above, [Ca^2+^]_*i*_ dynamics are also important to regulate actin cytoskeleton remodeling, which promotes CG movement toward the PM to release their content. The release of Ca^2+^ from the ER triggers the increase in the [Ca^2+^]_*i*_ and the binding of Ca^2+^ to calmodulin (Cam). It is a 17 kDa protein involved in multiple biological processes, including egg activation (Figure 1). In fact, Cam inhibitors induce a delay in meiosis resumption (Xu et al., 1996). One of the targets of Cam is myosin light chain kinase (MLCK) (Blumenthal et al., 1985). This kinase phosphorylates Myosin II, targeting either amino acids Ser19 or Ser19/Thr18 of its light chain (Colburn et al., 1988; Singer, 1990). Therefore, it promotes the binding of myosin II to actin filaments and CG translocation and spindle rotation (Matson et al., 2006). Ultimately, the inhibition of MLCK by ML-7 blocks CGE in mouse and human eggs (Lee et al., 2020). Another target of Cam is CamKII (Johnson et al., 1998; Tatone et al., 2002; Markoulaki et al., 2003). The activity of this kinase during egg activation oscillates following the periodicity of [Ca^2+^]_*i*_ increases (Markoulaki et al., 2004). The role of CamKII is important in CG traffic since eggs exposed to KN-93, an antagonist of this protein that inhibits CG release (Tatone et al., 1999).

In addition to unanchored CG from the actin cytoskeleton, it is necessary to control the rate of actin filaments polymerization to allow CGs to be prepared for exocytosis. It has been shown that MATER factor is critical in this step since there is no actin clearance in this protein null eggs (Figure 3B). In addition, the activity of myosin IIA is also required to depolymerize actin filaments before CGE (Vogt et al., 2019). Furthermore, stabilization of actin cytoskeleton by jasplakinolide prevents CG content release (Terada et al., 2000). These findings show and confirm that CGE is an actin remodeling-dependent process.

## Phylogenetic Distribution of Factors Regulating CGE During Egg Activation in Animals

**FIGURE 4:**
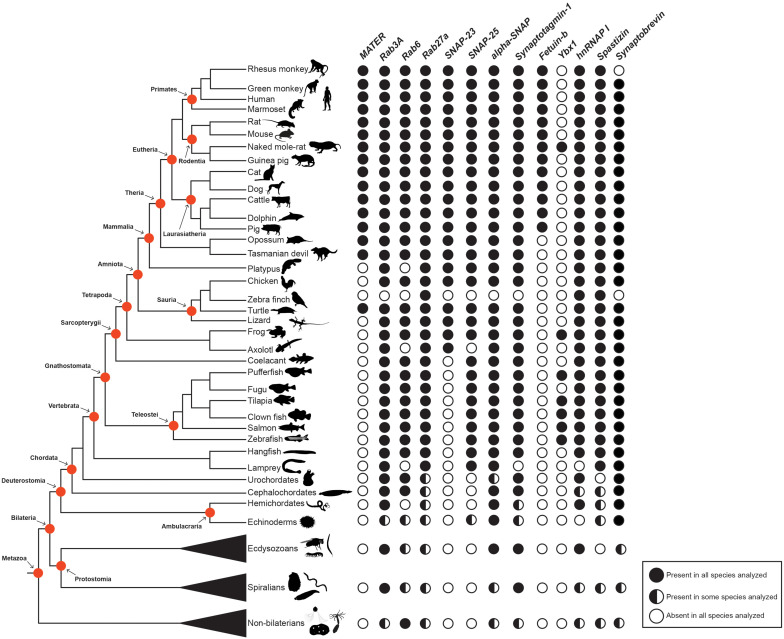
Phylogenetic distribution of molecular factors involved in cortical granule biology in animals. Protein presence or absence are indicated by black and white circles, respectively. Whilst proteins present in some, but not all, species surveyed are depicted by half-filled circles. Main taxonomy groups are indicated by red circles in the cladogram. The cladogram to the left represents the currently accepted phylogenetic relationships among animal taxa, focusing on vertebrate species. CGE, cortical granule exocytosis.

Currently, the use of animal models follows easy experimental protocols to isolate and manipulate the oocyte and egg. This represents an important tool to identify molecular factors involved in egg activation. In the previous sections, we have highlighted the function of a handful of these molecules, and indicated their roles in regulating CGE. From an evolutionary perspective, no information on the presence of these factors throughout animal phylogeny is reported. However, the availability of free access genome data from the main taxonomic groups of vertebrates and beyond (Dunn and Ryan, 2015), allows a survey to determine the phylogenetic distribution of these factors in animals (Figure 4).

This survey indicates that several molecular factors are well conserved across animal taxa. For instance, Rab proteins, which are known to play critical roles underlying cellular transport of vesicles (Martinez and Goud, 1998), are present in most species. Equivalently, SNARE complex proteins (i.e., SNAPs and synaptobrevin); a large protein superfamily comprising more than 60 members, can be found in several species (Figure 4) (Ungar and Hughson, 2003; Han et al., 2017). Additional molecular factors distributed widely in different taxa are Synaptotagmin-1 and Spastizin. The first, is a Ca^2+^ sensor located at the pre-synaptic axon terminal and responsible for triggering rapid exocytosis (Chapman, 2008). The latter is essential for the proper establishment of the motor neuron axonal network and CG maturation (Martin et al., 2012; Kanagaraj et al., 2014). In addition, hnRNAP I is also present in most animal species. It appears that some of the regulatory mechanisms underlying CGE during egg activation are shared in vertebrate and non-vertebrate species. However, this assertion needs to be further investigated because these proteins participate in a myriad of biological processes and in a variety of cell types.

Nonetheless, there are other molecular factors with a phylogenetic restricted distribution: Ovastacin, MATER, Feutin-b, and Ybx1 (Figure 4). This uneven distribution suggests that there are species-specific mechanisms underpinning the regulation of CGE in animals. Feutin-b and Ovastacin have a function in CG biology restricted to eutherians or placental mammals, indicating a possible co-evolution between these two proteins. Yet, their evolutionary history remains uninvestigated. Similarly, MATER is present in placental mammals, but also in marsupials, which suggests a possible conserved role of this protein across therian species. On the other hand, Ybx1 is restricted only to teleost species, with a putative role in CG accumulation (Sun et al., 2018). This species-specific distribution might be related to the evolutionary innovation of a chorion, the egg envelope in teleost species (Murata et al., 2014).

The phylogenetic distribution of molecular factors shown here (Figure 4) corresponds to a brief representation of the known group of proteins underpinning CGE in animal species. However, their actual contribution needs to be expanded in future investigations. Emerging molecular, physiology and phenogenomic tools are greatly impacting our understanding of reproductive biology. Therefore, we foresee that embracing the species comparative approach will answer long-standing questions about the evolution and fate of critical maternal factors and genetic control of CG biology.

## Discussion

The biogenesis and re-organization of the cellular organelles during the oocyte-to-embryo transition, including CGs, rely on the function of maternal factors and complex protein interactions. In this scenario, it is not surprising that the same cellular, molecular, and physiological principles controlling secretory vesicles biology are replicated in animal oocytes and eggs. Additionally, the use and combination of available experimental systems to study CG behavior, allows systematic multi-scale analysis of phenogenetic associations during oogenesis, egg activation, and fertility defects.

Egg activation triggers increase in [Ca^2+^]_*i*_ in all species studied to date (Kashir et al., 2013). CGE is Ca^2+^-dependent process and it is critical for polyspermy blockade in several animal species. Despite several years of investigations of these processes, surprisingly, little is known about the molecular actors orchestrating [Ca^2+^]_*i*_ rise.

Early events in development also involve other critical divalent ions. Interestingly, another ion that contributes to the ZP hardening is zinc (Zn^2+^). This cation is incorporated into the granules of the oocyte during its maturation (Que et al., 2015). These Zn^2+^-containing granules are located near the PM, and exocytosed after fertilization (Que et al., 2015). The release of this ion to the extracellular media is known as “Zn^2+^ sparks” and follows the Ca^2+^ oscillation patterns (Kim et al., 2011). For instance, Que et al. (2017) demonstrated that after fertilization, the Zn^2+^ concentration in the ZP increases by 300% and modulates its structure by augmenting its density. Moreover, when the ZP is exposed to this metal, the number of sperms interacting with the egg is reduced, indicating that ZP’s structural change caused by Zn^2+^ exposure is part of the polyspermy blockade mechanism (Que et al., 2017). These findings demonstrate a potential interplay between Ca^2+^ and Zn^2+^ to regulate secretory vesicle exocytosis and ZP hardening in the mammal egg.

TRPM7 also localizes in intracellular vesicles regulating Zn^2+^ release in somatic cells (Abiria et al., 2017). On the other hand, Mg^2+^ has been identified to be critical during early development, and TRPM7 channel is indicated to be critical player in Mg^2+^ homeostasis (Komiya et al., 2014). Moreover, conditional *Trpm7-*intestine deficient pups display high mortality by P10. Mutants are deficient in uptaking divalent cations, demonstrating the importance of these ions in early development. Dietary Zn^2+^ supplementation in *Trpm7-*intestine deficient mothers increases the survival curve of KO pups during pregnancy and breastfeeding (Mittermeier et al., 2019). Further, extracellular Mg^2+^ determines the frequency of Ca^2+^ oscillations during fertilization, most likely mediated by TRPM7 expression (Bernhardt et al., 2018). Future functional experiments in other vertebrate organisms will allow deciphering whether Zn^2+^, Mg^2+^, and other cations, physiologically control CG biology during egg activation and fertilization. This, will represent a significant advance in the knowledge of how to prepare the female gamete to start embryogenesis.

Maternally-loaded factors function in the transport, docking, and fusion. Such a function has also been described in other cell types. For example, the SNARE complex is critical for docking and fusion of neurotransmitters containing vesicles in neurons. At the cellular level, Spastizin is involved in intracellular trafficking and secretory vesicle maturation in the oocyte (Kanagaraj et al., 2014). Intriguingly, siRNA-mediated Spastizin knockdown displays a similar perturbed phenotype, revealing biological and physiological relevance of this factor in mammalian cells (Hirst et al., 2013). On the other hand, although fishes do not have ovastacin coding sequence, they do encode the astacin family protein alveolin, which is also involved in the hardening of the ZP (Shibata et al., 2012). This indicates that it is possible to track how conserved or lost functions during the oocyte-to-embryo transition and different reproductive strategies have evolved among animal species. Hence, the study of maternal-effect mutants and knockdown animals, displaying defects during CG biogenesis and exocytosis, will be highly informative for illuminating the function of maternal factors in the vertebrate oocyte and egg.

The cellular and molecular underpinnings of CG biology regulation can be comprehensively deciphered in vertebrate oocytes and eggs, which offer a myriad of advantages. These include easy experimental manipulation and culturing, optical properties, and single-cell analysis. Also, in the last decades, open access availability of complete genome sequences from different organisms has been pivotal to reveal the identity of key factors functioning during the oocyte-to-egg transition. Therefore, examining the one-cell female gamete by using high-throughput molecular and imaging phenotyping resources, will inform us about novel biological markers of reproduction and fertilization. In this way, zebrafish and mouse model systems can be integral to the study of vertebrate CG biology. This, will allow establish oocyte and egg quality selection criteria and potential therapies in human assisted reproductive technologies.

## Author Contributions

JR, FH, SV, IP-B, FA, RF, and IC contributed to conception and design of the article. IP-B and FA prepared the figures. IC and RF wrote the first draft of the manuscript. IC, RF, JR, FH, SV, and FA wrote sections of the manuscript. All authors contributed to manuscript revision, read, and approved the submitted version.

## Conflict of Interest

The authors declare that the research was conducted in the absence of any commercial or financial relationships that could be construed as a potential conflict of interest.

## Publisher’s Note

All claims expressed in this article are solely those of the authors and do not necessarily represent those of their affiliated organizations, or those of the publisher, the editors and the reviewers. Any product that may be evaluated in this article, or claim that may be made by its manufacturer, is not guaranteed or endorsed by the publisher.
